# Generation and Characterization of Conditional Heparin-Binding EGF-Like Growth Factor Knockout Mice

**DOI:** 10.1371/journal.pone.0007461

**Published:** 2009-10-14

**Authors:** Atsushi Oyagi, Yasuhisa Oida, Kenichi Kakefuda, Masamitsu Shimazawa, Norifumi Shioda, Shigeki Moriguchi, Kiyoyuki Kitaichi, Daisuke Nanba, Kazumasa Yamaguchi, Yasuhide Furuta, Kohji Fukunaga, Shigeki Higashiyama, Hideaki Hara

**Affiliations:** 1 Department of Biofunctional Evaluation, Molecular Pharmacology, Gifu Pharmaceutical University, Gifu, Japan; 2 Department of Pharmacology, Graduate School of Pharmaceutical Sciences, Tohoku University, Sendai, Japan; 3 Department of Pharmacology, Faculty of Pharmaceutical Sciences, Nagasaki International University, Nagasaki, Japan; 4 Department of Biochemistry and Molecular Genetics, Ehime University Graduate School of Medicine, Ehime, Japan; 5 Nihon Bioresearch Center Inc., Gifu, Japan; 6 Department of Biochemistry and Molecular Biology, The University of Texas M. D. Anderson Cancer Center, Houston, Texas, United States of America; Universidade Federal do Rio de Janeiro (UFRJ), Instituto de Biofísica da UFRJ, Brazil

## Abstract

Recently, neurotrophic factors and cytokines have been shown to be associated in psychiatric disorders, such as schizophrenia, bipolar disorder, and depression. Heparin-binding epidermal growth factor-like growth factor (HB-EGF) is a member of the EGF family, serves as a neurotrophic molecular and plays a significant role in the brain. We generated mice in which HB-EGF activity is disrupted specifically in the ventral forebrain. These knockout mice showed (a) behavioral abnormalities similar to those described in psychiatric disorders, which were ameliorated by typical or atypical antipsychotics, (b) altered dopamine and serotonin levels in the brain, (c) decreases in spine density in neurons of the prefrontal cortex, (d) reductions in the protein levels of the NR1 subunit of the *N*-methyl-D-aspartate (NMDA) receptor and post-synaptic protein-95 (PSD-95), (e) decreases in the EGF receptor, and in the calcium/calmodulin-dependent protein kinase II (CaMK II) signal cascade. These results suggest the alterations affecting HB-EGF signaling could comprise a contributing factor in psychiatric disorder.

## Introduction

Heparin-binding epidermal growth factor-like growth factor (HB-EGF) is a member of the EGF family of growth factors that includes EGF, transforming growth factor (TGF)-α, amphiregulin, betacelulin, and neuregulin [1], [2], [3]. HB-EGF binds to and activates the EGF receptor (EGF receptor/ErbB1) [1], ErbB4 [4], heparan sulfate proteoglycan (HSPG) [5], and N-arginine of dibasic convertase (NRDc) [6]. In the brain, EGF family members act as neurotrophic molecules, serving to enhance stem cell proliferation and neural differentiation, and they also influence synaptic plasticity [7], [8], [9]. HB-EGF is highly enriched in the neocortex and cerebellum [10], [11] and has been implicated in neuronal survival and glial/stem cell proliferation [12], [13], [14]. Recent studies indicate that this growth factor contributes to neurogenesis in the subventricular zone and adult hippocampus, thus maintaining multipotent neural progenitor cells expressing the ErbB1 receptor [13], [15]. HB-EGF also promotes the survival of dopaminergic neurons, an action mediated by mitogen-activated protein kinase (MAPK) as well as by the Akt signaling pathway [14]. For these reasons, HB-EGF may be an important contributor to neural development.

Abnormal development of the brain is implicated in the etiology and/or pathology of various psychiatric diseases, including schizophrenia [16], bipolar disorder [17], and depression [18], among others. Similarly, psychiatric patients can display abnormalities in the expression of cytokines and neurotrophic factors. For instance, the levels of brain-derived neurotrophic factor (BDNF) expression are specifically elevated in the hippocampus and cingulated cortex of schizophrenic patient, while conversely, its receptor, trkB protein, is reduced in the prefrontal cortex and hippocampus of them [19]. EGF protein levels have been found to be lower in the prefrontal cortex and striatum of schizophrenic patients [20]. Serum EGF levels were also lower in these patients, whereas EGF receptor expression in the prefrontal cortex was elevated [20]. All of these findings indicate that the EGF signal cascade may contribute to the pathology of a number of psychiatric diseases. On the other hand, since peptides other than authentic EGF (e.g., TGF-α and HB-EGF) are expressed at much higher levels than EGF itself [11], it is possible that HB-EGF serves as a major physiologic ligand for the EGF receptor within the central nervous system.

At the present time, little is known about the relationship between HB-EGF and psychiatric disorders. In the current study, therefore, we have generated HB-EGF conditional knockout (KO) mice that exhibit disruption of HB-EGF activity specifically in the ventral forebrain. We have compared the neurobehavioral and molecular biological characteristics of these mice with those of control mice and have demonstrated that the HB-EGF KO mice exhibit behavioral and biological abnormalities similar to those described in various psychiatric disorders. These results strongly suggest that alterations in HB-EGF signaling contribute to the pathogenesis of mental illness.

## Results

### Generation of HB-EGF KO mice

**Figure 1:**
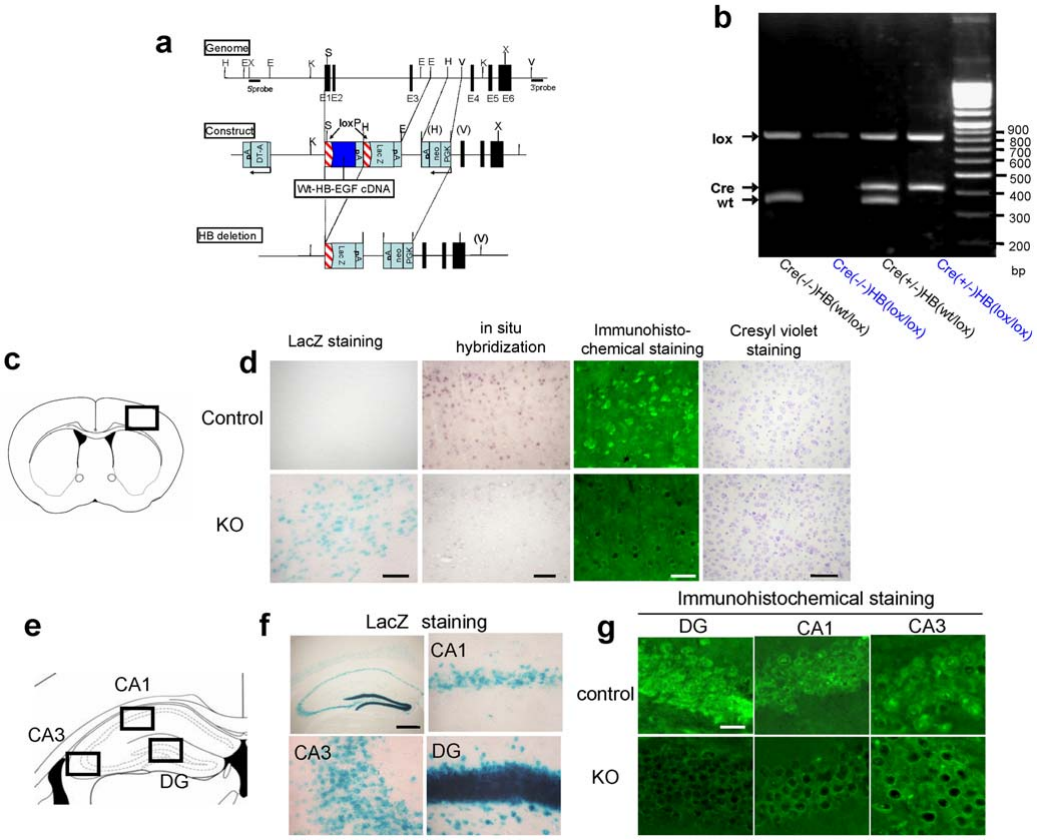
Generation of HB-EGF KO mice. (a) Gene-targeting strategy. Mouse *Hb-egf* cDNA containing the polyadenylation (pA) sequence flanked by loxP sequences was fused with the first exon of the mouse *Hb-egf* gene. The lacZ gene was inserted downstream of the *Hb-egf* cDNA. The targeting vector also contains the neomycin-resistance gene (neo), driven by the phosphoglycerate kinase (PGK) promoter and the diphtheria toxinA-fragment (DT-A) gene. Cre-mediated recombination results in the deletion of *Hb-egf* cDNA and in the expression of the lacZ gene. Exon sequences are indicated as black boxes. E, EcoR1; H, hind1; K, Kpnl; S, Sacll; V, EcoRV; X, Xhol. (b) Genotype of ventral forebrain-specific HB-EGF KO mice. Ventral forebrain-specific HB-EGF-deficient mice were confirmed by PCR as lox homozygous and Cre-recombinase positive. (c) Coronal section through the cortex region; square indicates area shown in the photomicrographs. (d) Histological analysis of cortex from controls (upper) and HB-EGF KO (lower) adult mice. LacZ staining, scale bar = 50 µm. In situ hybridization using an *Hb-egf* probe, scale bar = 100 µm. Immunohistochemical staining with anti-HB-EGF antibody, scale bar = 20 µm. Cresyl violet staining, scale bar = 500 µm. (e) Coronal section through the hippocampus; square indicates area shown in the photomicrographs (CA1, CA3, and DG). (f) LacZ staining of whole and individual hippocampal region in HB-EGF KO mice, scale bar = 500 µm. (g) Immunohistochemical analysis of individual hippocampal region from controls (upper) and HB-EGF KO (lower) adult mice, scale bar = 20 µm.

Since a homozygous null mutation of the *Hb-egf* gene causes lethality [21], in order to investigate the role of HB-EGF in the (adult/postnatal) brain, we generated ventral forebrain specific HB-EGF KO mice using a Cre-lox-mediated conditional gene KO approach with Six3 promoter [22]. This strategy for generating the HB-EGF KO mice, hereafter referred to as Cre (+/−) HB (lox/lox), is shown in Fig. 1a. The resulting Cre (+/−) HB (lox/lox) and control HB (lox/lox) mice were identified by polymerase chain reaction (PCR) analysis (Fig. 1b). HB (lox/lox) mice showed the same expression patterns for HB-EGF mRNA and protein in various regions of the brain (hippocampus, prefrontal cortex, cerebellum, and striatum) when compared with wild-type C57BL/6J mice (data not shown). Additionally, we confirmed the disruption of *Hb-egf* gene by lacZ staining. LacZ positive cells were founded in the prefrontal cortex and hippocampus in KO mice (Fig. 1d, and f). For a further examination of the expression of *Hb-egf* mRNA and its protein in HB-EGF KO and control mice, we performed in situ hybridization analysis and immunohistrochemical analysis respectively (Fig. 1d). In the prefrontal cortex, the expression of *Hb-egf* mRNA and protein were found to be considerably reduced in KO mice (Fig. 1d). Similarly, HB-EGF protein was undetectable beyond the background level in the cell layer of CA1, CA3, and dentate gyrus (DG) of KO mice hippocampus (Fig. 1g). However, we could detect no significant differences in the weights of individual brain regions (Table S1), and cresyl violet staining of adult brain sections showed no difference in the lamination or structure of cortical cells between HB-EGF KO mice and their control littermates (Fig. 1d). There were no significant differences in the body weight of adult KO or control mice. Mean body weights of mice used for each experiment are as follows: Locomotor activity and social interaction [Control; 27.0±0.51 g (mean±S.E.M., n = 25), KO; 26.6±0.52 g (n = 22)], PPI [Control; 25.5±0.33 g (n = 53), KO; 24.9±0.38 g (n = 65)], Monoamine analysis [Control; 25.2±0.69 g (n = 11), KO; 25.8±0.73 g (n = 11)], Y-maze, Novel object recognition test, and Western blotting [Control; 22.0±0.63 g (n = 6), KO; 22.0±0.73 g (n = 6)], and Morphological analysis [Control; 22.4±0.59 g (n = 5), KO; 21.6±0.74 g (n = 5)].

### HB-EGF KO mice exhibited abnormal psychomotor behaviors

**Figure 2:**
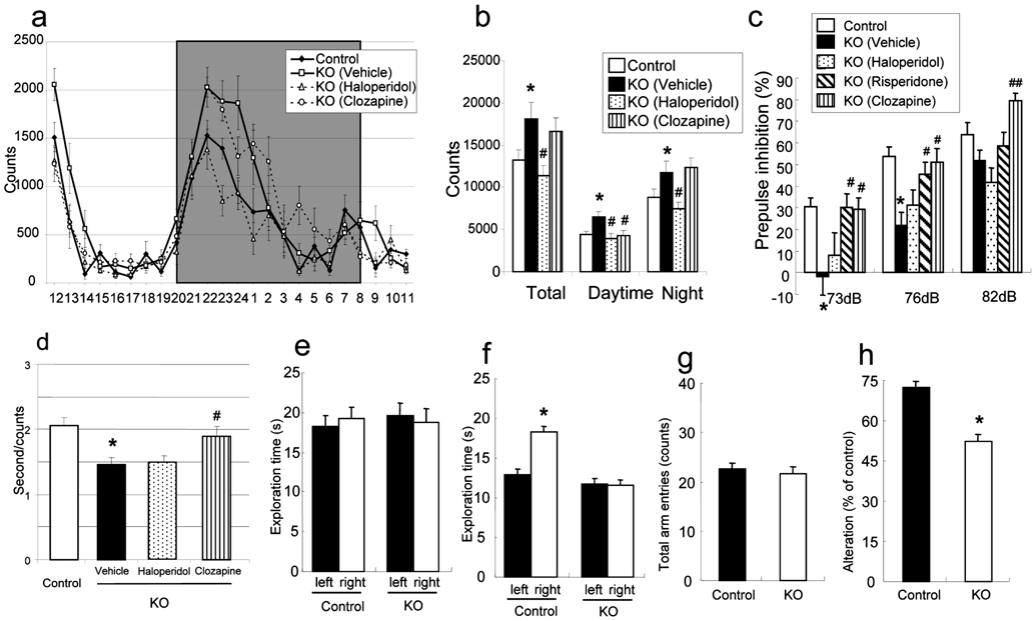
Behavioral analysis of HB-EGF KO mice and control mice. (a and b) Locomotor activity test. Mice were placed into individual cages, and their locomotion was assessed every hour for 1 day. Controls (n = 8), KO mice with treatment of vehicle (n = 7), haloperidol (n = 8), and clozapine (n = 8). (a) Locomotor activity throughout the 24-hr period, and (b) locomotor activity analyzed separately during day and night periods. (c) PPI of the acoustic startle response in control (n = 24), KO mice with treatment of vehicle (n = 24), haloperidol (n = 15), risperidone (n = 14), and clozapine (n = 12). The PPI is expressed as a percentage of the startle response to a 120 dB pulse. (d) Social interaction test in a novel environment in controls (n = 8) and KO mice with treatment of vehicle (n = 7), haloperidol (n = 9), and clozapine (n = 9). Two genetically identical mice that had been housed separately were placed in the same cage. Their social interaction was then monitored for 10 min. Values are means±SEM. (e and f) Novel-object recognition task. (e) In the training trial (5 min), two circles were placed in symmetrical left and right positions. (f) In the test trial 1 hr later (5 min), one circle (left) and one triangle (right) were placed in the same positions. The amount of time the control (n = 6) and KO (n = 6) mice spent exploring each object during training trial and test trial was recorded. * p<0.05 vs. left object. (g and h) Y-maze task. Each mouse was placed at the end of one fixed arm of the maze and allowed to move freely through the maze for 8 min. The sequence of arm entries was recorded manually. (g) Total number of arms entered during the session. (h) % of alternation was calculated as (actual alternations/maximum alternations) ×100. Control (n = 6), KO (n = 6). Values are means±SEM. * p<0.05 vs. control mice. #p<0.05, ##p<0.01 vs. vehicle-treated HB-EGF KO mice.

To investigate the involvement of HB-EGF in psychomotor behaviors, we performed several types of neurobehavioral tests. First, we studied the behavior of HB-EGF KO and control mice in their cages to measure locomotor activity. HB-EGF KO and control mice displayed a normal circadian rhythm (i.e., their activity was reduced during the light phase [8 A.M. to 8 P.M.] and then increased markedly at the beginning of the dark phase) (Fig. 2a). However, HB-EGF KO mice were more active than control mice over the 24-hr period (both dark and light phases) (P<0.05 vs. control mice) (Fig. 2a and b). During the first 0–3 hr of the test, HB-EGF KO mice were also more active than control mice, indicating that HB-EGF KO mice are also hyperactive in a novel environment. To test the involvement of dopaminergic and serotonergic neuronal systems in the increased locomotor activity, we examined the effects of a potent dopamine (DA) receptor antagonist, haloperidol, and a DA/serotonin (5-HT) receptor antagonist, risperidone and clozapine, in HB-EGF KO mice. Haloperidol (0.1 mg/kg, i.p.) ameliorated the hyperlocomotion of HB-EGF KO mice both in dark and light phases (P<0.05 vs. vehicle-treated HB-EGF KO mice in each phases). On the other hand, clozapine reduced the locomotor activity of HB-EGF KO mice only in light phase. Furthermore these drugs did not attenuate locomotor activity in control mice at each dose (Table S2).

To investigate the possible role of HB-EGF in sensorimotor gating, prepulse inhibition (PPI) was measured in HB-EGF KO and control mice. Sensorimotor gating is the neural process in which allow attention to be focused on one stimulus. Sensorimotor gating can be assessed as PPI of startle, which is the modulation of the startle response, following a weak prepulse. In the pulse-only trials, startle amplitude did not differ significantly between HB-EGF KO and control mice, [Control; 263.7±33.56 (mean±S.E.M, n = 25), KO-vehicle; 205.8±35.25 (n = 24), KO-haloperidol; 230.2±60.6 (n = 15), KO-risperidone; 212.5±43.78 (n = 14), and KO-clozapine; 225.2±35.03 (n = 12)]. HB-EGF KO mice showed diminished PPI at prepulse intensities of both 73 and 76 dB (P<0.05 vs. control mice) (Fig. 2c). Moreover, PPI deficit observed in HB-EGF KO mice were significantly reversed by atypical antipsychotics, risperidone and clozapine (P<0.05 vs. vehicle-treated HB-EGF KO mice), but not by a typical neuroleptic haloperidol (P>0.05). These drugs did not affect PPI in control mice at each dose (Table S2).

To assess the social affiliative behavior of HB-EGF KO mice, we employed a social interaction paradigm. When introduced to another mouse, a normal mouse will typically investigate the new mouse by walking over and sniffing it. But animal models of psychiatric disorders tend to show decreases in such social-interaction behavior. During a 10 min social interaction test in a novel environment, the mean duration per contact was significantly less in HB-EGF KO mice than in control mice, indicating that HB-EGF KO mice spent significantly less time engaging in social contact (P<0.05 vs. control mice) (Fig. 2d). We could not detect any differences in olfactory sensation between either genotype (data not shown). Mice were pretreated with haloperidol or clozapine for 7 days and subjected to the social interaction test. Clozapine, but not haloperidol, significantly attenuated the decreased social interaction behavior of HB-EGF KO mice (P<0.05 vs. vehicle-treated HB-EGF KO mice) (Fig. 2d). During a 10 min social interaction test, not only the mean duration per contact but also the time of interaction were significantly less in HB-EGF KO mice than in control mice [Control; 41.7±3.37 sec (mean±S.E.M, n = 8), KO-vehicle; 27.1±5.13* sec (n = 7), KO-haloperidol; 21.0±2.68 (n = 9), KO-clozapine; 24.7±2.44 (n = 9), *p<0.05 vs. control mice]. On the other hand, no significant changes were observed in the number of interactions between HB-EGF KO and Control mice [Control; 20.4±1.31 counts (mean±S.E.M, n = 8), KO-vehicle; 18.4±2.72 counts (n = 7), KO-haloperidol; 14.7±1.86 (n = 9), KO-clozapine; 13.2±1.31 (n = 9)]. Furthermore these drugs did not affect the social interaction in control mice at each dose (Table S2).

Using a novel-object recognition test, we next investigated the effect of deletion of the *Hb-egf* gene on learning and memory performance. The exploratory behavior of the HB-EGF KO mice and control mice did not differ during the training session (P>0.05 vs. control mice) (Fig. 2e). However, in the test trials conducted 1 hr after the training session, control mice spent significantly more time exploring a new object than a familiar object, exhibiting a clear preference for the novel object (P<0.05 vs. control mice) (Fig. 2f). In contrast, HB-EGF KO mice spent equal amounts of time investigating the novel object and the familiar object (P>0.05 vs. control mice) (Fig. 2f).

Next, to further assess the specificity of changes in working memory related to the loss of HB-EGF protein, we elected to test spontaneous alternation in the Y-maze, which is devoid of any procedural bias because it is based on the natural tendency of mice to explore a novel environment. There was no difference in the number of arm choices or in preference for a particular arm between HB-EGF KO and control mice (Fig. 2g). However, HB-EGF KO mice displayed a significant decrease in alternation compared to control mice (P<0.05 vs. control mice) (Fig. 2h).

### Changes in monoamine levels

**Figure 3:**
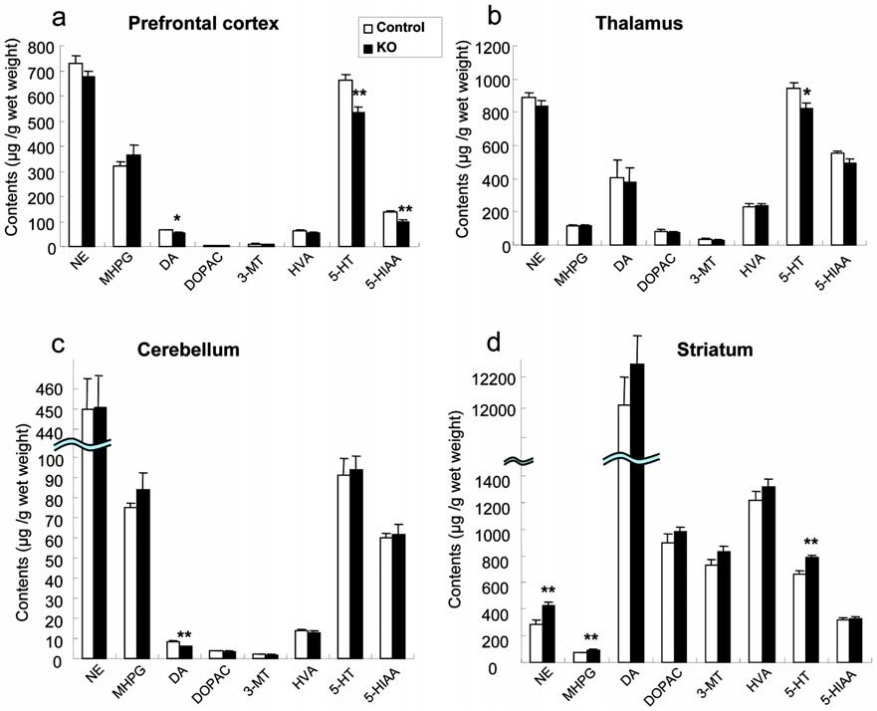
Tissue monoamine levels of individual brain regions. Levels of monoamine neurotransmitters and their major metabolites were assayed in the (a) prefrontal cortex, (b) thalamus, (c) cerebellum, and (d) striatum of controls (n = 11) and HB-EGF KO (n = 11) mice. Values are means±SEM. * p<0.05, ** p<0.01 vs. control mice. NE: Norepinephrine, MHPG: 3-methoxy 4-hydroxy phenethyleneglycol, DA: Dopamine, DOPAC: 3,4-dihydroxyphenilacetic acid, 3-MT: 3-methoxytyramine, HVA: homovanillic acid, 5-HT: Serotonin, 5-HIAA: 5-hydroxyindol acetic acid.

It is commonly believed that behavioral abnormalities are associated with alterations in monoaminergic transmission [23]. To investigate whether targeted disruption of the *Hb-egf* gene affected the function of monoaminergic neuronal systems, tissue levels of monoamines and their metabolites were assessed in various regions of the brain in HB-EGF KO and control mice. In the prefrontal cortex, DA, 5-HT, and its metabolite 5-hydroxyindole acetic acid (5-HIAA) levels were statistically lower in HB-EGF KO mice than in control mice (P<0.05 vs. control mice) (Fig. 3a). The 5-HT level in the thalamus and the DA levels in the cerebellum were also lower in HB-EGF KO mice than in control mice (P<0.05 vs. control mice) (Fig. 3b and c). In contrast, the striatal norepinephrine (NE), its metabolite 3-methoxy-4-hydroxyphenylglycol (MHPG), and 5-HT levels were higher in HB-EGF KO mice than in control mice (P<0.05 vs. control mice) (Fig. 3d). On the other hand, no significant differences in monoamine metabolites in various regions of the brain (prefrontal cortex, thalamus, cerebellum, and striatum) were observed between HB-EGF KO and control mice (Figure S1).

### Morphological changes in the prefrontal cortex

**Figure 4:**
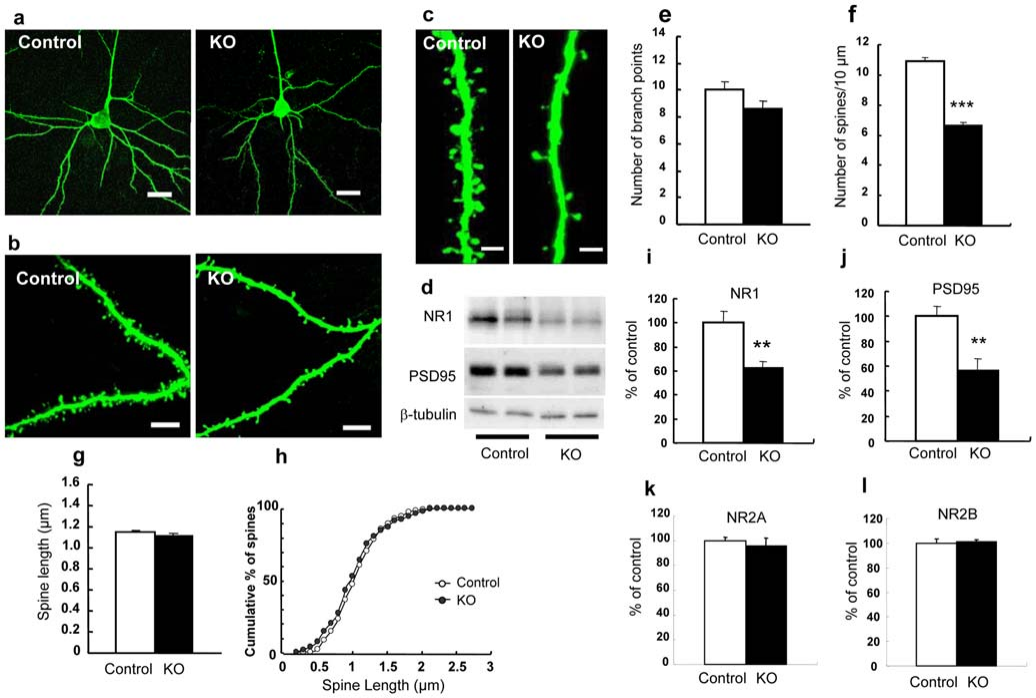
Morphological changes in the prefrontal cortex. (a) Representative photomicrographs showing morphology of pyramidal neurons in cortical layer III of the prefrontal cortex from control (left) and KO (right) mice. Scale bar = 20 µm. (b) Representative photomicrographs of apical dendritic segments from control (left) and KO (right) mice. Scale bar = 8 µm. (c) High-magnification images of apical dendritic segments from adult control (left) and KO (right) mice. Scale bar = 2 µm. (d) Representative images of immunoblots showing NR1 and PSD-95 protein levels. (e) Quantification of the number of basal dendritic branch-points. (f) Spine density on primary apical dendrites of layer III pyramidal neurons of the prefrontal cortex from control (white bar) and KO (black bar) mice. (g) Quantification of spine length. Control (n = 700 spines) and KO (n = 592 spines) mice (n = 4 mice, 25 neurons each). (h) Cumulative distribution of spine length. Control (white circle) and KO (black circle) mice (n = 4 mice, 25 neurons each). (i, j, k, and l) Quantitative analysis of NR1, PSD-95, NR2A, and NR2B by densitometric scanning of immunoreactive bands. Control (n = 5), KO (n = 5). Data for control and KO mice are expressed as a percentage of the control value. Values are means±SEM. ** p<0.01, *** p<0.001 vs. control mice.

To try to identify possible morphological correlates of the observed abnormalities of behavior and cognition, we analyzed the morphology of pyramidal neurons in cortical layer III in the prefrontal cortex. Fig. 4a–c shows the morphology of such neurons in control (left) and HB-EGF KO (right) mice. In these neurons, (I) there was no significant difference in the number of basal dendritic branch-points between controls and HB-EGF KO mice (P>0.05 vs. control mice) (Fig. 4a and e); (II) as regards spine density in primary apical dendrites, the number of spines per 10 µm of dendritic segment was lower in KO than in control mice (P<0.0001 vs. control mice) (Fig. 4b, c, and f); (III) there was no significant difference in dendrite length between adult controls and KO mice (P>0.05 vs. control mice) (Fig. 4g); and (IV) as regards the cumulative distribution of spine length, there was no significant difference between adult controls and KO mice (P>0.05 vs. control mice) (Fig. 4h).

Next, we assessed by western blotting the protein levels of NR1 and PSD-95 as the obligatory subunits of the NMDA receptor and its interacting molecule, which are thought to be involved in the pathophysiology of schizophrenia. Fig. 4d shows immunoreactive bands for NR1 and PSD-95. Both NR1 and PSD-95 were markedly reduced in the prefrontal cortex of HB-EGF KO mice (P<0.01 vs. control mice) (Fig. 4d, i, and j). In contrast, no significant differences were observed in the expression of NR2A and total NR2B proteins between controls and HB-EGF KO mice (P>0.05 vs. control mice) (Fig. 4k and l).

### Dysfunction of CaMKII and its related signals in HB-EGF KO mice

**Figure 5:**
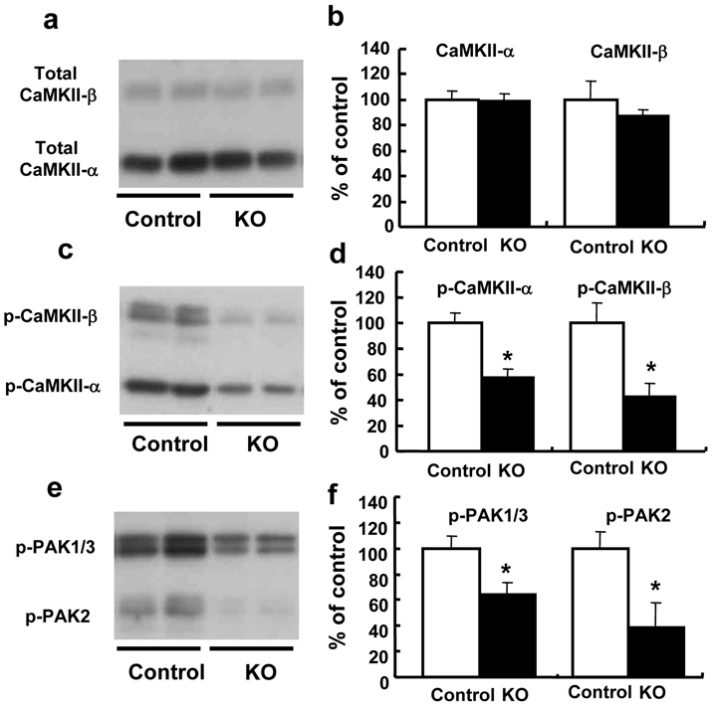
Activation of CaMKII and its related signals in HB-EGF KO and control mice. Representative images of immunoblotting showing total CaMKII (a), p-CaMKII (c), p-PAK1/3, and p-PAK2 (e). Quantitative analysis of total CaMKII (b), p-CaMKII (d), p-PAK1/3, and p-PAK2 (f) by densitometric scanning of immunoreactive bands. Data for control (n = 5) and KO (n = 5) mice are expressed as a percentage of the control value. Values are means±SEM. * p<0.05 vs. control mice.

By using western blotting, we next evaluated the protein levels of total CaMKII α and β, phosphorylated CaMKII (p-CaMKII) α and β, p21-activated kinase (p-PAK)1/3, and p-PAK2, which are serine-threonine kinases that regulate the synaptic architecture in neurons. Fig. 5a and c show immunoreactive bands for total CaMKII and p-CaMKII, respectively. P-CaMKII (α and β) was markedly reduced in the prefrontal cortex of HB-EGF KO mice (P<0.01 vs. control mice) (Fig. 5d), despite the presence of a normal level of total CaMKII protein (Fig. 5b). Likewise, protein levels of p-PAK1/3 and p-PAK2 were significantly lower in the prefrontal cortex in HB-EGF KO mice than in control mice (P<0.01 vs. control mice) (Fig. 5e and f).

### Expression of the EGF receptor and its signals

**Figure 6:**
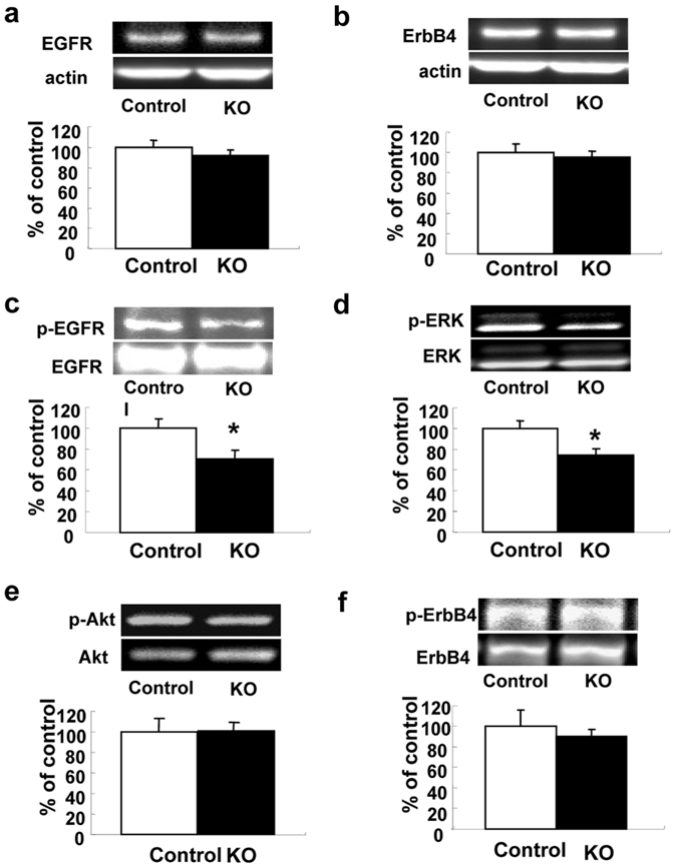
Expression of EGF receptor and its signals in HB-EGF KO and control mice. Representative images of immunoblots showing EGF receptor and β-actin (a), ErbB4 and β-actin (b), p-EGF receptor and total EGF receptor (c), p-ERK, ERK, and β-actin (d), p-Akt, Akt, and β-actin (e), and p-ErbB4 and ErbB4 (f) in the prefrontal cortex (upper). Quantitative analysis of EGF receptor (a), ErbB4 (b), p-EGF receptor (c), p-ERK (d), p-Akt (e), and p-ErbB4 (f) by densitometric scanning of immunoreactive bands, with normalization to the signals for β-actin (a and b), EGF receptor (c), ERK (d), Akt (e), or ErbB4 (f) (lower). Data for control (n = 5) and KO (n = 5) mice are expressed as a percentage of the control value. Values are means±SEM. * p<0.05 vs. control mice.

To investigate the mechanisms that might lead to the observed behavioral abnormalities and altered monoamine levels, we compared the expression of the EGF receptor and its signaling in HB-EGF KO and control mice. No significant differences in the expression of total EGF receptor or ErbB4 proteins were observed between controls and HB-EGF KO mice (P>0.05 vs. control mice) (Fig. 6a and b). Despite these equivalent levels of EGF receptor expression, p-EGF receptor protein was significantly decreased in the prefrontal cortex of HB-EGF KO mice (P<0.05 vs. control mice) (Fig. 6c). Moreover, the expression of phosphorylated extracellular signal-regulated kinase (p-ERK) protein was significantly decreased in the prefrontal cortex of HB-EGF KO mice (P<0.05 vs. control mice), despite comparable levels of total ERK expression (Fig. 6d). No significant differences in the expression of phosphorylated and total Akt or ErbB4 proteins were observed between controls and HB-EGF KO mice (P>0.05 vs. control mice) (Fig. 6e and f). On the other hand, we did not detect significant differences between controls and KO mice regarding the relative expression of other EGF family growth factors, such as EGF [Control; 100±4.58 (mean±S.E.M., n = 10), KO; 104±6.94 (n = 10)], TGF-α [Control; 100±11.8 (n = 10), KO; 104±9.36 (n = 10)], and betacelulin [Control; 100±6.59 (n = 10), KO; 99.6±6.48 (n = 10)], using real-time PCR. No Real-time PCR activity for epiregulin mRNA was observed under our experimental conditions in either HB-EGF KO or control mice.

## Discussion

In this study, we generated conditional HB-EGF KO mice using a Cre/loxP system and were able to confirm the disruption of HB-EGF in the prefrontal cortex and hippocampus of KO mice. These results corresponded with a previous report which detected the Six3 expression in the ventral forebrain and basal ganglion [24]. Despite the decreased expression HB-EGF, the overall development of HB-EGF KO mice appeared to be normal and there were no significant differences in the body weight in the adult KO or control mice. We also detected no significant differences in the weights of individual brain regions, and adult brain sections showed no difference in the lamination or structure of cortical cells when investigated in either HB-EGF KO mice or their control littermates.

To investigate the roles of HB-EGF in psychomotor behavior, we performed a comprehensive behavioral analysis of ventral forebrain-specific HB-EGF KO mice. Deficiencies in PPI and memory tasks are considered to be signs of cognitive dysfunction [25], whereas social withdrawal is one of the most disabling symptoms in psychiatric disorders such as depression and schizophrenia [26]. Hyperactivity is a characteristic of the rodent models of several psychiatric disorders, such as schizophrenia [27] and attention deficit hyperactivity disorder (ADHD) [28] and could correspond to psychomotor agitation observed in human patients with these disorders. The behavioral impairments observed in HB-EGF KO mice were similar to those observed in mice with treatment of NMDA receptor antagonists such as phencyclidine (PCP) [29], [30], [31]. Consistent with these reports, in the present studies, treatments with either a typical or an atypical antipsychotic drug ameliorated these behavioral impairments in KO mice. A typical antipsychotic (haloperidol) reduced the locomotor activity of KO mice, but not the social withdrawal or the deficit of PPI. In contrast, atypical antipsychotics (clozapine and risperidone) ameliorated the impairments of social interaction and PPI.

HB-EGF KO mice also displayed deficit of memory related behavior, as observed by the Y-maze test performance and novel-object recognition test. These deficits are considered to be signs of cognitive and working memory dysfunction, respectively [32], [33]. Taken together, the results from the present study indicate that HB-EGF KO mice present with a comprehensive spectrum of psychomotor and cognitive dysfunctions, all of which are characteristic of rodent models of psychiatric disorders, such as schizophrenia.

In general, abnormal behaviors are accompanied by altered monoamine levels in animal models of psychiatric disorders [23]. Contrary to the classical DA hypothesis, which proposes that hyperactivity of DA transmission is responsible for schizophrenia [34], the DA level was statistically lower in the prefrontal cortex of the HB-EGF KO mice than in the control mice. However, a recent DA hypothesis of schizophrenia also indicates a decrease in DA level in the prefrontal cortex [35]. HB-EGF is known to promote the survival of midbrain dopaminergic neurons [14]; therefore, the absence of an *Hb-egf* gene might be expected to result in hypoplasia and hypofunction of dopaminergenic neurons. On the other hand, the upregulation of NE, 5-HT, and its metabolites observed in the striatum of the KO mice in the present study may compensate for the downregulation of the prefrontal monoamine levels.

The presence of NMDA receptor hypofunction in psychiatric disorder has been inferred recently from a large number of clinical and preclinical observations [36]. Genetic experiments in animals suggest that the NR1 subunit, an essential part of the NMDA receptor, plays an important role in cognitive function [37], [38]. In the present study, NR1 protein was markedly reduced in the prefrontal cortex of HB-EGF KO mice. Malfunctioning NMDA receptor signaling in the prefrontal cortex of KO mice may lead to dysfunction in this brain region, perhaps resulting in the impairments of recognition and spatial working memory that were observed in this study.

The most significant finding made in the present study was that the HB-EGF KO mice had a significantly decreased spine density on the primary apical dendrites of the layer III pyramidal neurons of the prefrontal cortex (vs. control mice) of approximately 40%. Since the dendritic spine is the major site of excitatory synaptic transmission in the central nervous system, the size and density of these spines will clearly influence the operation of functional circuits [39], [40], [41]. In schizophrenic patients, spine density of these neurons is decreased compared with normal controls [42]. The KO mice also showed decreased expression of the NR1, PSD-95, p-CaMKII, and PAK families, and these signaling pathways are known to regulate synaptic structure and function in the developing cortex [43], [44], [45]. Thus, the morphological changes observed in dendritic spines may have been a result of the observed decreases in the expression of the NR1, PSD-95, p-CaMKII, and PAK families. These in turn may have led to the abnormalities in behavior and monoamine levels seen in the KO mice.

Additionally, current evidence indicates that cytokines and growth factors can strongly influence neural phenotypic differentiation and subsequent neural function. Some studies have indicated a potential link between impaired EGF signaling and the pathogenesis of schizophrenia [20], [46]. The EGF receptor is expressed ubiquitously in both the developing and the adult brain [47], [48]. However, since peptides other than authentic EGF (e.g., TGF-α and HB-EGF) are expressed at much higher levels than EGF itself [11], HB-EGF itself could be serving as a major physiologic ligand for EGF receptor within the central nervous system. In the present study, we detected a decrease in p-EGF receptor protein and subsequent activation of ERK signaling in the prefrontal cortex of HB-EGF KO mice (vs. control mice). Consistent with our results, a previous study (using the same line of mice with loxP-flanked *Hb-egf* cDNA) demonstrated that a mutation of the *Hb-egf* gene was associated with decreases in the activation of EGF receptor and ERK [49].

MAPKs, also known as ERKs, operate in a signaling cascade that regulates various cellular processes (such as proliferation, differentiation, and cell cycle progression). ERKs are recognized as being important signaling components in learning and memory and in regulating dendritic spine density [50]. Current evidence indicates that continued exposure to clozapine stimulates p-ERKs, mediated by the EGF receptor [51]. Down-regulation of EGF receptor signaling, induced by mutation of the *Hb-egf* gene, could affect synaptic plasticity by regulating NDMA relating proteins, and this would lead to the abnormal behaviors observed in HB-EGF KO mice.

In conclusion, the current study demonstrates that HB-EGF KO mice exhibited the behavioral abnormalities reflected in a comprehensive spectrum of psychomotor and cognitive dysfunctions, similar to many psychiatric disorders. Based on the observations from the current study, we proposed a role for HB-EGF in determining monoaminergic neural system and synaptic plasticity. Many psychiatric disorders are multifactorial, reflecting longitudinal and complex interactions of causative agents, including genetic and environmental factors. These findings indicate that the HB-EGF signaling may play a pivotal role in the pathogenesis of a number of psychiatric disorders, including schizophrenia.

## Materials and Methods

### Generation of HB-EGF KO mice using a gene targeting Cre-loxP and PCR

The targeting strategy has been described previously [21]. The targeting construct is depicted in Fig. 1a. A 7.0-kb EcoRI–SacII fragment containing exon 1 of the HB-EGF gene, a 1.3-kb EcoRI–HindIII fragment from intron 3, and a 6.0-kb EcoRV fragment downstream of the exon 4 were used as homology arms. Mouse HB-EGF cDNA and a poly (A) signal flanked by loxP were fused at exon 1. A 4.0-kb HindIII–XhoI fragment containing the loxP-lacZ gene-poly (A) signal was inserted downstream of the HB-EGF cDNA. A neo cassette driven by the phosphoglycerate kinase promoter was inserted between the 1.3-kb intron 3 EcoRI–HindIII fragment and the 6.0-kb EcoRV fragment downstream of exon 4. XhoI-linearized DNA of the targeting vector was electroporated into TT2 embryonic stem (ES) cells. Individual clones were screened for homologous recombination by Southern blot analysis of HindIII-digested DNA, corresponding to sequences that flank the targeting vector 5′- and 3′-arm used in the screening of the HBlox allele. The targeted ES clones were injected into ICR blastocysts, and the resulting chimeric mice were bred with C57BL/6J female mice to obtain HB (lox/wt) mice. Homozygous HB (lox/lox) mice were then obtained by interbreeding the HB (lox/wt) mice. Homozygous HB (lox/lox) mice (C57BL/6J) were bred with Six3 promoter driven by Cre-recombinase transgenic mice (C57BL/6-) [22] to generate Six3-Cre-HB (lox/wt) mice. Subsequently, Six3-Cre-HB (lox/wt) mice were bred with HB (lox/lox) mice to generate Six3-Cre-HB (lox/lox) mice (HB-EGF KO mice) and HB (lox/lox) mice (HB-EGF control mice). The genotype of each mouse was confirmed by PCR. The primers have been reported previously [21]. Amplification was performed using a DNA thermal cycler (PERKIN ELMER, Norwalk, CT, USA) for 35 cycles. A cycle profile consisted of 30 sec at 94°C for denaturation and 30 sec at 53°C for annealing and primer extension. In the present study, to minimize the influence of different genetic backgrounds between HB (lox/lox) mice (C57BL/6J) and Six3-Cre transgenic mice (C57BL/6-), the generated Six3-Cre-HB (lox/wt) mice were backcrossed onto C57BL/6J HB (lox/lox) mice for more than 8 generations. HB (lox/lox) mice (C57BL/6J) used for backcrossing were maintained by inbred mating.

### Histological analysis

Mice (male, 10–20 weeks old) were anesthetized with sodium pentobarbital (nembutal, 50 mg/kg, i.p., Dainippon Pharmaceutical, Osaka, Japan), and the brains were perfusion-fixed with 4% paraformaldehyde (Wako Pure Chemical Industries, Osaka, Japan) in 0.1 M phosphate-buffered saline (PBS) (pH 7.4). The brains were removed from the mice after 20 min of perfusion fixation at 4°C and then immersed in the same fixative solution. Brain sections were equilibrated in 25% sucrose solution and quickly frozen in Tissue-Tek O.C.T. (Sakura Finetek, Torrance, CA, USA).

For lacZ staining, after fixation with 0.2% glutaraldehyde and 1% formalin, tissues were incubated with 5-bromo-4-chloro-3-indoly-β-galactoside (X-Gal) (Active Motif, Carlsbad, CA, USA) for 2 hr at 4°C. For immunohistochemical staining, frozen sections were washed for 5 min in 0.01 M PBS and then treated with 0.3% hydrogen peroxidase in 10% methanol. They were then washed three times in 0.01 M PBS, followed by a 30 min pre-incubation with 10% normal goat serum. They were then incubated with a rat anti-HB-EGF antibody (1∶200), including 0.3% triton X-100, overnight at 4°C. After a 15 min rinse in changes of 0.01 M PBS, the sections were incubated with a secondary antibody, Alexa 488-conjugated goat anti-rat IgG (Molecular Probes, Carlsbad, CA, USA). In situ hybridization analysis was performed using an antisense DIG-labeled cRNA probe. DIG-labeled cRNA probes corresponding to *Hb-egf* (NM_010415) nucleotide positions 546 to 665 were synthesized, in situ hybridization analysis was performed, as previously reported [52], and frozen sections (10 µm thick) were stained with cresyl violet (Sigma, St. Louis, MO, USA).

### Behavioral tests

All mice (male, 10–15 weeks old) were housed in a room with a 12-hr light/dark cycle (light on at 8:00 a.m.) and had ad libitum access to food and water. Behavioral tests were performed between 9:00 a.m. and 5:00 p.m., except for the 24-hr locomotor activity test. All procedures relating to animal care and treatment conformed to the Animal Care Guidelines of the Animal Experiment Committee of Gifu Pharmaceutical University.

### Locomotor activity test

To measure the locomotor activity, a mouse was placed in the cage, [plastic cage (175×245×125 mm), wood-chips, food, and water]. Locomotion was measured every 1 hr for 1 day using digital counter with infrared sensor (NS-ASS01; Neuroscience, Inc, Tokyo, Japan). Animals were placed into the cages at 2:00 P.M. for a 24 period. The room light was on from 8:00 A.M. to 8:00 P.M. Vehicle (0.3% tartaric acid), clozapine (1.0 mg/kg, i.p.), or haloperidol (0.1 mg/kg, i.p.) was administrated once a day for 9 days, and one day after the final injection, locomotor activity test was started.

### Prepulse inhibition (PPI) test

Acoustic startle responses were measured in a startle chamber (SR-LAB; San Diego Instruments, San Diego, CA) using standard methods described previously [53]. In the demonstration of PPI of acoustic startle reflex, subjects were presented with a series of discrete trials comprising a mixture of four types of trials. These included pulse-alone trials, prepulse-plus-pulse trials, prepulse-alone trials, and trials in which no discrete stimulus, other than the constant background noise, was presented. A reduction of startle magnitude in prepulse-pulse-pulse trials relative to those in pulse-alone trials constitutes PPI. The pulse stimulus employed was 120 dB in intensity and 40 msec in duration. Prepulse of various intensities as employed: 73, 76, and 82 dB. The duration of prepulse stimuli was 20 msec. The stimulus onset asynchrony of the prepulse and pulse stimuli on prepulse-plus-pulse trial was 100 msec. A session began with the animals being placed into the Plexiglas enclosure. Clozapine (WAKO) was dissolved in 0.1N HCl in saline and neutralized to pH 6–7 with 0.1N NaOH. Haloperidol (WAKO) and risperidone (WAKO) were dissolved in distilled water containing 0.3% tartaric acid. Vehicle (0.3% tartaric acid), clozapine (1.0 mg/kg, i.p.) haloperidol (0.1 mg/kg, i.p.), and risperidone (0.1 mg/kg, i.p.) were administrated 30 min before the measurement of PPI. Mice were acclimatized to the apparatus for 5 min before the first trial began. Subsequently the animals were presented with 3 blocks of discrete test trials. Each block consisted of one trial of each of the following trial types: pulse-alone, prepulse-plus-pulse trials of each of the five levels of prepulse, prepulse-alone of each of the five levels of prepulse, and no stimulus (ie background alone). The interval between successive trials was variable with a mean of 30 sec (ranging from 20 to 40 sec).

### Social interaction test

The social interaction test in a novel environment was done in a manner similar to published method [54]. Each mouse was housed in a group of 5–6 animals of the same genotype per cage during the test. Two mice of identical genotypes, which were previously housed in different cages, were placed into a box together (175×245×125 mm) and allowed to explore freely for 10 min. Social behavior was monitored by a video camera. The number and mean duration of contacts for 10 min after the start were measured. During the 10 min observation period, observers monitored the following behaviors: social interaction, sniffing, following, genital investigation, facing, and mounting. The score was calculated as the mean duration per contact. Haloperidol (0.1 mg/kg, i.p.) and clozapine (1.0 mg/kg, i.p.) were administrated once a day for 7 days, and one day after the final injection, social interaction test was started.

### Novel object recognition task

Mice were tested in a circular arena, 40 cm in diameter. All mice were habituated in the apparatus (without objects) for 40 min per day prior to the training session. At the end of each trial, the animals were removed from the arena and the arena was cleaned with 70% ethanol solution and dried with paper toweling. Object recognition was scored by the amount of time spent for each object, with the nose of the mouse directed to and located within 2 cm from the object or with the anterior limbs touching the object. In a first trial (T1: 5 min) two similar objects (left and right: circle) were placed in a symmetrical position 5 cm away from the wall. In a second trial (T2: 5 min), two plastic dissimilar objects [one with a circle (left) and a new one triangle (right)] were presented. The number of account exploring each object during T1 and T2 was recorded. All mice were tested 1 hr between T1 and T2.

### Y-maze task

Recording the spontaneous alteration behavior in Y-maze was assessed as spatial reference memory task. The apparatus consisted of three identical arms (50 l×16 w×32 h cm) made by black Plexiglas. Each mouse was placed at the end of one fixed arm and allowed to move freely through the maze during an 8 min session. The sequence of arm entries was recorded manually. An alternation was defined as entries into all three arms on consecutive choices. The number of maximum alternations was then the total number of arms entered minus two, and the percentage of alternation was calculated as (actual alternations/maximum alternations) ×100. In addition, the total number of arms entered during the session was also determined.

### Measurements of monoamine neurotransmitters and metabolites

Each mouse (male, 10–20 weeks old) was decapitated under deep anesthesia and its brain quickly removed from the skull, briefly washed in ice-cold saline, and laid on a cooled (4°C) metal plate, on which it was rapidly dissected to separate the prefrontal cortex, striatum, cerebellum, and thalamus. The dissected brain regions were weighed, frozen, and stored at −80°C until analysis. The regional levels of monoamines and their metabolites were determined using a high-performance liquid chromatography (HPLC) system equipped with an electrochemical detector (HPLC-ECD System; Eicom, Kyoto, Japan). The HPLC system and conditions were as follows: Eicom EP-300/Eicom ECD-300 system; column, Eicompak SC-5ODS (3.0 mm i.d.×15 cm) with precolumn; mobile phase, 83% 0.1 M citric acid/0.1 M sodium acetate buffer, 17% methanol, 0.18% sodium 1-octanesulfonate, containing 5 mg/ml disodium-EDTA; flow rate, 0.5 ml/min; electrode, Eicom WE-3G graphite electrode; reference electrode, Eicom RE-100 Ag/Ag Cl; applied voltage, 750 mV versus Ag/Ag Cl. Column temperature was maintained at 25°C.

### Morphological analysis

Mice were anesthetized with pentobarbital and perfused via the ascending aorta with phosphate-buffered saline (PBS; pH 7.4) until the outflow became clear, followed by 0.1 M phosphate buffer (PB; pH 7.4) containing 4% paraformaldehyde (PFA) for 15 min. Brains were removed and postfixed in the same fixative for 24 h at 4°C. The brain was sliced at 150 µm thickness using a vibratome (Dosaka EM Co. Ltd, Kyoto, Japan). The slices were transferred into a Silgard-coated Petri dish filled with 4′, 6-diamidino-2-phenylindole (DAPI) solution (1 µg/ml; Sigma, St. Louis, MO) in PBS. Neurons in the infralimbic (IL) and prelimbic (PL) regions of the medial prefrontal cortex (mPFC) were penetrated with glass microelectrodes filled with 3% LY (lithium salt; Sigma) in distilled water, and LY was injected by passing 2–20 nA current with 2.5 second pulses applied at 0.2 Hz for 1–2 minutes. After LY injections, sections were postfixed in the same fixative for 1–2 days, and then were incubated as follows: several washes in PBS, 1 h in 0.1 M PBS containing with 1% BSA, 0.3% TritonX-100 and 0.1% NaN3 (blocking solution); and with a rabbit anti-LY polyclonal antibody (1∶5000, Molecular probes) in blocking solution at 4°C for 4–5 days. After washing, sections were incubated for 3 h with Alexa 488-labeled anti-rabbit IgG (1∶500, Molecular probes). After several washes in PBS, sections were mounted on slides with Vectashield (Vector Laboratory). Immunofluorescent images were analyzed using a confocal laser scanning microscope (Leica TCS). Pyramidal neurons within the infralimbic and prelimbic regions were defined by the presence of a single apical dendrite. For analyses of branching, basal dendrites were evaluated by counting the number of branches on each dendrite. Data were expressed as the mean number of branches per dendrite. The average spine density (number of spines per 10 µm of dendritic length) was estimated on the first segments of apical dendrites. For analyses of spine density and spine length, spines were identified on the basis of the morphological criteria for ‘mushroom’ and ‘thin’ spines described by Peters and Kaiserman-Abramof [55], with the only protrusions counted being those perpendicular to the dendritic shaft and possessing a clear neck and bulbous head. Spine length represents the radial distance from the tip of the spine head to the dendritic shaft. These morphological analyses were performed using 6 to 8 neurons each from five controls and HB-EGF KO mice.

### Immunoblotting analysis

Immunoblotting analyses of the prefrontal cortex region were performed using male, 10-week-old animals (controls and KO mice). Extracts prepared from the medial prefrontal cortex region were treated with Laemmli's sample solution and boiled for 3 min. Samples containing equivalent amounts of protein were separated on 10% sodium dodecyl sulfatepolyacrylamide gels and transferred for 2 hr at 70 V to a polyvinylidene difluoride membrane using the method of Towbin et al [56]. Membranes were then incubated for 1 hr in blocking solution containing 4% dry milk in T-TBS (20 mM Tris-HCl, pH 7.5, 150 mM NaCl, and 0.1% Tween 20) at room temperature and then incubated overnight at 4°C with antibodies against PSD-95 (1∶250, Transduction Laboratories, Lexington, KY, USA), NR1, NR2A (1∶1000, Upstate Biotechnology, Lake Placid, NY, USA), Nr2B (R&D Systems, Minneapolis, MN, USA), EGF receptor, ErbB4 (1∶1000, Santa Cruz Biotechnology, Santa Cruz, CA, USA), phospho-CaMKII (p-Thr286/p-Thr287) (1∶5000), anti-CaMKII (1∶5000),β-tubulin (1∶5000) ERK, p-ERK, AKT, p-AKT, PLCγ, p-PLCγ, phospho-PAK1 (Thr423), phospho-PAK2 (Thr402), or phospho-PAK3 (Thr421) (1∶1000, Cell Signaling Technology, Beverly, MA, USA) in blocking solution. After washing in T-TBS, membranes were incubated with horseradish peroxidase-conjugated anti-mouse or anti-rabbit IgG to visualize primary antibodies using enhanced chemiluminescence plus western blotting detection reagent (Amersham Pharmacia Biotech, Amersham, UK), according to the manufacturer's protocol. The images were scanned and analyzed semi-quantitatively using NIH Image (Research Services Branch of the National Institute of Mental Health). Optical densities of corresponding immunoreactive bands were obtained by subtraction of the background density within the same image.

### Autophosphorylation of ErbB receptor in HB-EGF KO mice

Immunoblotting analyses of the autophosphorylation of the ErbB receptor were performed using the prefrontal cortex region. In this study, membrane protein was extracted using a Native Membrane Protein Extraction Kit (Calbiochem, Cambridge, MA, USA) according to the manufacturer's instructions. Then, membrane protein was immunoblotted using an anti-phospho-EGF receptor antibody and an anti-phospho-ErbB4 antibody (1∶1000, Cell Signaling Technology), as described above.

### Statistical analysis

All data were expressed as the mean±SEM. Statistical significant was evaluated either by either one-way or two-way ANOVA test followed by a post hoc Dunnett's test. A *P*-value of <0.05 was considered to be statistically significant.

## Supporting Information

Figure S1 Supporting figures
(0.16 MB DOC)

Table S1 Supporting Table
(0.04 MB DOC)

Table S2 Supporting Table
(0.02 MB DOC)
