# Supplementary material for: Generation and Characterization of Conditional Heparin-Binding EGF-Like Growth Factor Knockout Mice
Source: PLoS One. 2009 Oct 14;4(10):e7461. doi: 10.1371/journal.pone.0007461 (PMC2759290; doi:10.1371/journal.pone.0007461)
Supplement: Figure S1 — Supporting figures (0.16 MB DOC) [file pone.0007461.s001.doc]

**Supplemental figure**


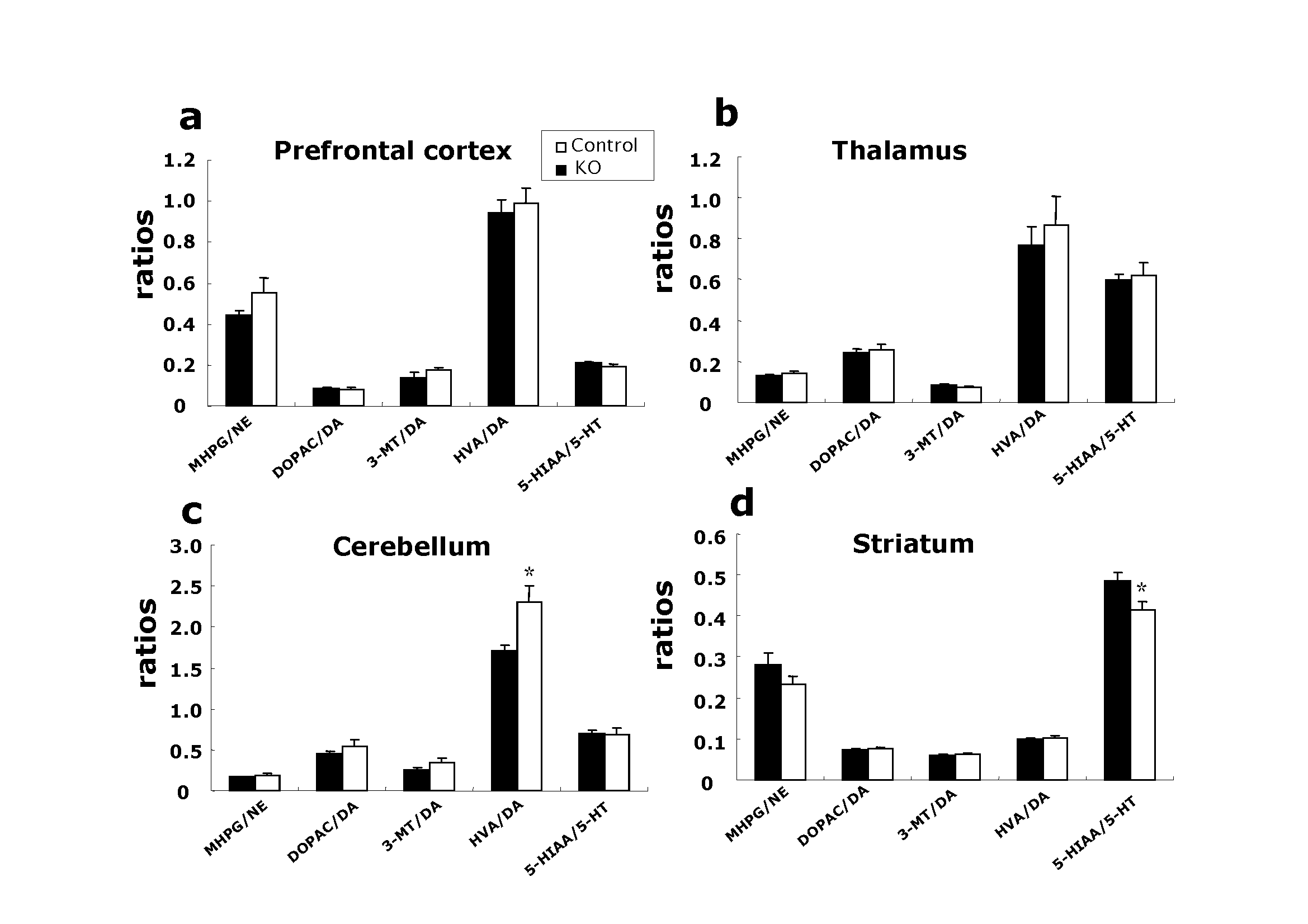


**Fig. S1. Monoamine turnover in HB-EGF KO and control mice.**

Individual MHPG/NE, DOPAC/DA, 3-MT/DA, HVA/DA, and 5-HIAA/5-HT ratios in the (**a**) prefrontal cortex, (**b**) thalamus, (**c**) cerebellum, and (**d**) striatum of control (n=11) and HB-EGF KO (n=11) mice. Values are means ± SEM. * p < 0.05 vs. control.
