# Supplementary material for: Generation and Characterization of Conditional Heparin-Binding EGF-Like Growth Factor Knockout Mice
Source: PLoS One. 2009 Oct 14;4(10):e7461. doi: 10.1371/journal.pone.0007461 (PMC2759290; doi:10.1371/journal.pone.0007461)
Supplement: Table S1 — Supporting Table (0.04 MB DOC) [file pone.0007461.s002.doc]

**Table S1. Weight of individual brain regions in control and HB-EGF KO mice.**

**Total brain weight**

**Prefrontal**

**cortex**

**Medial**

**frontal cortex**

**Striatum**

**Thalamus**

**Cerebellum**

470±10

12.63±1.01

11.25±1.30

19.51±1.64

33.80±2.69

55.53±1.76

(100)

(100)

(100)

(100)

(100)

(100)

470±10

10.91±0.67

12.39±1.03

17.16±1.30

32.14±1.42

54.90±1.34

(100)

(86.4)

(110.1)

(95.1)

(98.9)

**Control**

**KO**

(88.0)

Values (mg) represent the means ± SEM (Control: n=11, KO: n=11). Numbers within the parentheses indicate the percentage of the weight relative to that of the same region in Control mice.
