# Supplementary material for: Generation and Characterization of Conditional Heparin-Binding EGF-Like Growth Factor Knockout Mice
Source: PLoS One. 2009 Oct 14;4(10):e7461. doi: 10.1371/journal.pone.0007461 (PMC2759290; doi:10.1371/journal.pone.0007461)
Supplement: Table S2 — Supporting Table (0.02 MB DOC) [file pone.0007461.s003.doc]

**Table S2. Effect of antipsychotics on locomotor, PPI, and social interaction in control mice.**

Values represent the means ± SEM. Numbers within the parentheses indicate the number of mice used.
